# Formula Milk Supplementation on the Postnatal Ward: A Cross-Sectional Analytical Study

**DOI:** 10.3390/nu10050608

**Published:** 2018-05-14

**Authors:** Kirsty V. Biggs, Katherine Hurrell, Eleanor Matthews, Ekaterina Khaleva, Daniel Munblit, Robert J. Boyle

**Affiliations:** 1 Brighton and Sussex Medical School, Brighton BN2 5BE, UK; K.Biggs1@uni.bsms.ac.uk; 2 Royal United Hospital, Combe Park, Bath BA1 3NG, UK; Katherine.hurrell@nhs.net; 3 Department of Paediatrics, Imperial College Healthcare NHS Trust, London W2 1NY, UK; ellie.matthews@imperial.nhs.uk (E.M.); daniel.munblit08@imperial.ac.uk (D.M.); 4 Department of Paediatrics, Saint-Petersburg State Paediatric Medical University, 194353 Saint-Petersburg, Russia; doctor.khaleva@gmail.com; 5 inVIVO Planetary Health, Group of the Worldwide Universities Network (WUN), 6010 Park Ave, West New York, NJ 07093, USA; 6 Faculty of Pediatrics, I. M. Sechenov First Moscow State Medical University, 119991 Moscow, Russia

**Keywords:** breastfeeding, attitudes, knowledge, midwifery, formula supplementation, justification of supplementation

## Abstract

Breastfeeding rates are low in the UK, where approximately one quarter of infants receive a breastmilk substitute (BMS) in the first week of life. We investigated the reasons for early BMS use in two large maternity units in the UK, in order to understand the reasons for the high rate of early BMS use in this setting. Data were collected through infant feeding records, as well as maternal and midwife surveys in 2016. During 2016, 28% of infants received a BMS supplement prior to discharge from the hospital maternity units with only 10% supplementation being clinically indicated. There was wide variation in BMS initiation rates between different midwives, which was associated with ward environment and midwife educational level. Specific management factors associated with non-clinically indicated initiation of BMS were the absence of skin-to-skin contact within an hour of delivery (*p* = 0.01), and no attendance at an antenatal breastfeeding discussion (*p* = 0.01). These findings suggest that risk of initiating a BMS during postnatal hospital stay is largely modifiable. Concordance with UNICEF Baby Friendly 10 steps, attention to specific features of the postnatal ward working environment, and the targeting of midwives and mothers with poor educational status may all lead to improved exclusive breastfeeding rates at hospital discharge.

## 1. Introduction

Breastfeeding has long been regarded as the preferred infant feeding choice with established long- and short-term benefits for mothers, infants, and society [1,2]. Many high-quality studies from developed countries similar and applicable to the United Kingdom (UK) show that breastfed babies are less likely to develop infections than babies fed with breastmilk substitutes (BMS), specifically those of the lower respiratory tract, gastrointestinal system, and otitis media [3,4,5,6,7,8,9]. Indeed, a meta-analysis of 33 studies in developed counties concluded formula-fed infants had three times more severe infections compared to infants exclusively breastfed to four months [10].

Studies have found other beneficial health associations of breastfed infants compared to infants fed with BMS, including lower rates of Sudden Infant Death Syndrome, necrotizing enterocolitis, atopic diseases, childhood obesity, and enhanced neurocognitive function [11,12,13,14,15,16,17,18,19,20].

Breastfeeding not only confers health benefits to the infant, but also to the mother, for example by incurring faster weight loss and as a natural contraceptive [21,22]. The longer the duration of breastfeeding, the increased protection against breast and ovarian cancer, and reduced post-menopausal hip fractures [23,24,25,26,27]. Emerging evidence suggests that breastfeeding positively impacts mother-baby relationships, bonding, and post-natal depression [28,29,30]. From an economic perspective, the improvements in child and maternal morbidity and mortality, plus reduced workforce productivity loss are estimated to be substantial [1].

Exclusive breastfeeding (EBF) is defined as an infant who receives only breastmilk, or expressed breastmilk, but no other solids or liquids except prescribed medicines, vitamins, or mineral supplements [31].

The World Health Organisation (WHO) recommends all infants to be breastfed exclusively for the first six months of life, and for breastfeeding to continue until age 2 years or beyond [31]. Despite this, data from the National Infant Feeding survey 2010 demonstrates <1% of mothers in the UK reach this target. The greatest decline in exclusive breastfeeding (23%) occurred within the infant’s first week of life, with infants who exclusively breastfed for the first two days having a greater chance of achieving the six-month target, with greater self-efficacy [32,33,34]. This demonstrates the importance of providing adequate in-hospital support.

The literature has highlighted associations between the use of BMS and negative impacts on breastfeeding. In a prospective cohort study of mothers with the intention to exclusively breastfeed, the introduction of BMS on post-natal wards, alongside intrapartum opiate-use, were the only modifiable predictors of non-exclusivity at three months [35]. Chantry et al. found a three-fold increase in the risk of breastfeeding cessation by day 60 for those receiving in-hospital BMS supplementation, regardless of the mother’s intention [36]. There are also concerns about BMS use and its impact on the neonatal gut microbiota, with long-term health implications [37]. Current evidence has demonstrated that early in-hospital supplementation is commonplace in developed countries [34].

The use of BMS supplementation within the early hospital environment is a multifactorial practice. The most commonly cited reason for BMS supplementation was due to breastfeeding difficulties, demonstrating that a high proportion of supplementation occurs without medical explanation [38]. Significant variation in BMS supplementation of breastfed infants was also reported in concordance with maternal education and ethnicity [39]. Within the early postnatal environment, midwives are responsible for the initial administration of supplementary BMS. Previous work has identified a lack of time, poor staffing levels, and a resistance to change as key contributing factors to non-compliance of a breastfeeding support intervention on UK postnatal wards [40].

The training a midwife receives has also been suggested to be clinically relevant to infant feeding practices in the hospital, with those demonstrating higher breastfeeding knowledge reporting best clinical practice [41]. A UK comparative study identified negative staff attitudes as a contributing factor to the early cessation of breastfeeding [42]. However, qualitative research suggests that midwives underestimate the influence they have on maternal feeding choices [43,44]. Overall, these studies highlight the need to investigate the influence of maternal and midwife factors on rates of neonatal infant-formula supplementation in the absence of medical indication (NIFS-AMI).

## 2. Materials and Methods

### 2.1. Study Design

**Figure 1:**
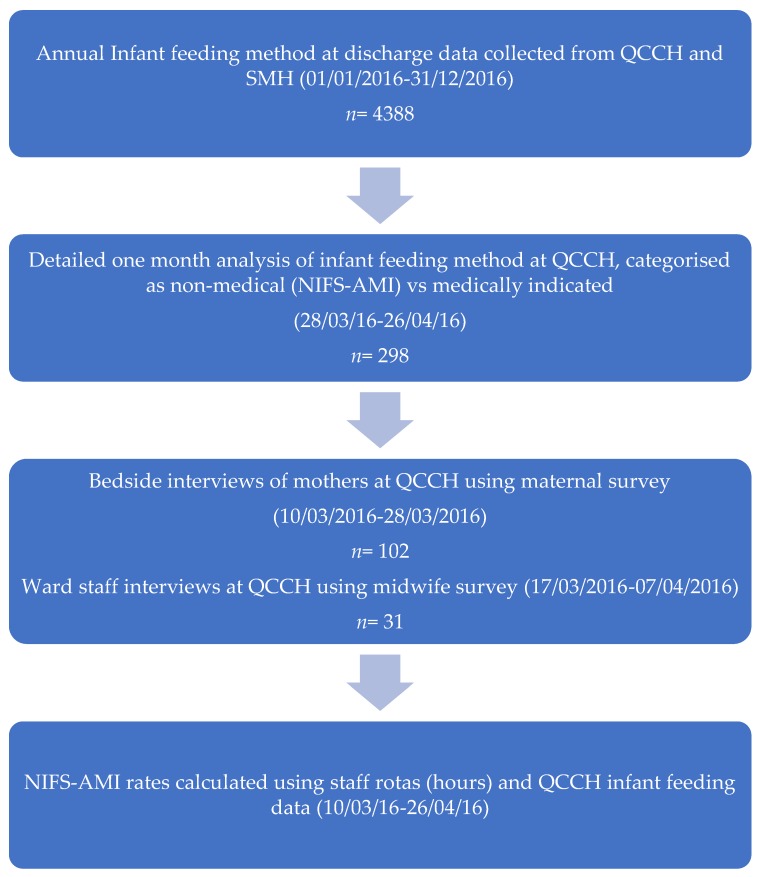
Study design. QCCH: Queen Charlotte’s and Chelsea Hospital; SMH: Saint Mary’s Hospital; NIFS-AMI: neonatal infant-formula supplementation in the absence of medical indication.

A cross-sectional analytic study evaluated features of mothers, midwives, and postnatal care which are associated with NIFS-AMI on the postnatal ward (Figure 1). Data were routinely collected over one year about feeding method at discharge at Queen Charlotte’s and Chelsea Hospital (QCCH) and St Mary’s Hospital (SMH), UK to determine the recorded supplementation rate within the Trust. A detailed analysis of one month’s data from the hospital’s patient records determined infant feeding method and further categorisation of supplementation into either medical or non-medical reasons (NIFS-AMI). Bedside interviews of mothers were conducted to determine maternally reported supplementation rate and explore the relationship between features of birth, mother, and family and risk of NIFS-AMI. Interviews of midwives were used to determine the range and values of midwife NIFS-AMI rate and explore midwife characteristics associated with this rate.

### 2.2. Study Participants

We undertook two separate cross-sectional surveys. In the maternal survey, mothers of newborn infants were surveyed at the bedside between 10 March 2016 and 28 March 2016. Women with conditions or medications that prevented breastfeeding, who had multiple pregnancies, or whose infants were inpatients on the neonatal intensive care unit were excluded. All participating mothers gave informed consent.

In the midwife survey, midwives working on the postnatal wards between 17 March 2016 and 7 April 2016 were assessed. Royal College of Midwifery registered midwives working at least one shift during this period, either as agency, temporary, or permanent staff members, were included. The postnatal department is divided into three distinct wards; the public National Health Service (NHS) facility (43 beds), the self-pay (private) ward (eight private rooms), and the NHS birth centre (eight birthing rooms). Care is primarily delivered by midwives and maternal support workers. At the study site, BMS cannot be initially administered without the involvement and approval of a registered midwife. An obstetrician may be present on the private and NHS postnatal wards; however, the birth centre is an entirely midwife-led provision for labour and post-natal care. As part of the NHS screening programme, an in-hospital newborn and infant physical examination (NIPE) is conducted on all neonates by a paediatrician prior to discharge. Typically in the UK, mother-infant dyads are discharged to the community midwifery team once assessed as healthy, having established feeding and having received appropriate education on caring for their infant. Home visits are provided by community midwives for the first 10 days until care transitions to the responsibility of the health visitor. A general practitioner will provide a six-week postnatal check.

All participating midwives provided verbal consent and were assured that their responses would remain anonymous. They were informed of the purpose of the study—to examine behaviours associated with formula supplementation. The project was conducted as a clinical audit, as part of the requirements of the Baby Friendly Initiative. Formal review by an ethics committee was therefore not required, but the audit was approved by the clinical department and was approved by the Queen Charlotte’s and Chelsea Hospital, London, UK audit office (March 2016).

### 2.3. Data Collection

Data were collected through infant feeding records, as well as maternal and midwife surveys. Annual infant feeding data were retrieved from CERNER electronic patient records, demonstrating the feeding method at discharge for 4388 mother-infant dyads at QCCH and SMH during the study period (1 January 2016 to 31 December 2016). Data from SMH were included to better assess the generalisability of the participants recruited from QCCH. Length of admission, ethnicity, delivery methods, parity, birth weight, gestation, and ward type were compared between the two hospital sites. To determine infant feeding method at inpatient discharge, data from ward records on the postnatal unit were collected from 298 patients between 28 March 2016 and 26 April 2016. CERNER medical and nursing notes for both mother and infant were accessed to assess any recorded medical reasons for supplementation. Reasons for supplementation were categorised as either medical or non-medical (NIFS-AMI), according to WHO criteria. Accepted clinical reasons for using BMS included the following infant conditions: classic galactosemia, maple syrup urine disease, and phenylkenonuria. Infants who may require BMS in addition to breastmilk for a limited period include very low birth weight (<1500 g), very pre-term (<32 weeks gestation), and newborn infants who are at risk of hypoglycaemia if their blood glucose fails to respond to optimal breastmilk feeding. Maternal conditions that justify the temporary avoidance of breastfeeding included severe illness that prevents a mother from caring for her infant (e.g., sepsis), herpes simplex virus type 1 lesions on the mother’s breasts, and certain maternal medications. HIV infection was the only named justification for permanent avoidance of breastfeeding [45].

#### 2.3.1. Maternal Survey

All participating women were interviewed with the UNICEF Baby Friendly Hospital Initiative (BFHI) accredited audit tool [46]. Data were collected with regard to the education received on breastfeeding and relationship building from a healthcare professional, their birth experience, and the antenatal and postnatal support received. Original additional questions were included regarding maternal demographic data, infant feeding methods, durations of breastfeeding both as a mother and as an infant, and general attitudes of their close friends and relatives. Maternal levels of breastfeeding (BF) confidence were assessed using a Likert scale (1–10) for both before and after the birth of their infant. Maternal demographic data, gestation at delivery, and infant birth weight were obtained from patient electronic records. Ethnicity was categorised according to Office of National Statistics England [47].

#### 2.3.2. Midwife Survey

Quantitative and qualitative data were obtained through staff questionnaires to assess attitudes as well as personal and professional experiences of infant feeding. The survey instrument was developed based on expert opinion and previously published literature [40,41,42,43,44]. Within the questionnaire, staff were asked to describe three scenarios in which they had supplemented a breastfed infant and to discuss the barriers they encountered with regard to supporting breastfeeding.

We also obtained data on the frequency of initiated NIFS-AMI across the three wards and for each midwife. Infants supplemented for medical reasons were excluded from analysis. To control for the inevitable influence of working hours, a supplementation rate was calculated. Staff rotas from the corresponding seven-week period were reviewed to total the number of hours each midwife worked, which was divided by the supplementation frequency to produce an hourly percentage rate. The hourly rate was used to estimate a monthly rate; the hourly rate was multiplied by 37.5 and divided by 7 before multiplying the value by 30.

### 2.4. Data Analysis

Data were tested for normality to determine the choice of statistical methods using a visual inspection of histograms and the Shapiro-Wilk test. For univariate analysis, the Mann-Whitney U test for non-parametric data was employed. For categorical data, Pearson’s Chi-squared test was used to determine statistically significant differences between groups. Fisher’s Exact Test was used if cells had an expected count of less than 5. A *p*-value of <0.05 was considered significant. The Spearman Correlation coefficient was used to test associations between variables with a non-parametric distribution.

Due to the relatively small sample size, the private ward and birth centre were grouped due to similarities in size, facilities, staff to patient ratios, and working environment when using ‘postnatal ward type’ as a variable in analyses.

Analysis of covariance (ANCOVA) was used for multivariate analysis, considering cofounding variables that had been identified through correlation coefficients and a review of the literature, to produce the final model.

IBM SPSS 23 statistical software (IBM, Armonk, NC, USA) was used for all statistical analysis.

## 3. Results

### 3.1. Maternal Descriptive Characteristics

Table 1 presents the clinical and demographic characteristics of mothers and infants surveyed, in comparison to QCCH and SMH electronic records during April 2016. Of the 102 women included in the study, 55 were exclusively breastfeeding (EBF) their baby (54%), 41 were supplementing with BMS (40%), and six were exclusively receiving BMS (6%). The monthly rates of exclusive breastfeeding at discharge for 2016 are displayed in Figure S1. The median maternal age was 32 years (28, 36 IQR) and infant age was 1 day (1, 2). Primiparous women were overrepresented in our study sample (58%); however, other characteristics were illustrative of the wider cohort. 

### 3.2. Maternal Characteristics and NIFS-AMI

Univariate analysis was conducted to determine which variables of maternal characteristics were associated with NIFS-AMI (Table 2). Skin-to-skin contact for at least one hour (*p* = 0.01), higher education (*p* < 0.01), and antenatal discussions regarding breastfeeding (*p* = 0.01) were all inversely associated with non-clinical supplementation behaviour on the postnatal ward.

### 3.3. Midwife Descriptive Characteristics

The demographics of the participating midwives are detailed in Table S1. The median age was 38 years (28–50 IQR) with the majority having studied midwifery at the university level (87%). The median years’ experience as a midwife was 7 years (4–18). All the participating staff members had received mandatory breastfeeding training provided by Imperial College London NHS Hospital Trust (100%). Of the 31 midwives available for interview, 20 had initiated NIFS-AMI within the seven-week study period (65%), with a median hourly rate of 0.62% (0–2.8).

### 3.4. Midwife Attitudes and Recalled Infant Feeding Behaviour

Twenty-four midwives reported their cultural and social backgrounds to have a positive attitude towards breastfeeding (77%) (Table 3). The mean perceived impact of BMS supplementation was 8/10 (6–10), with 1 being lowest impact and 10 greatest. Of all the supplementation scenarios described by staff, 62% (*n* = 111) violated WHO criteria, with only a third able to describe three scenarios appropriately and 13% unable to provide any acceptable answers. Inappropriate reasons cited for BMS supplementation are shown as number (%); hungry infant 15 (21.7); medical recommendation 13 (18.8); jaundice 12 (17.4); latching problems 10 (14.5); low birth weight or weight loss 8 (11.6); prematurity 7 (10.1); reluctant feeders 1 (1.5); nasogastric tube-use 1 (1.5); transitional care 1 (1.5); 12 h without feeding 1 (1.5).

### 3.5. Assessment of Real-Life BMS Prescription

**Figure 2:**
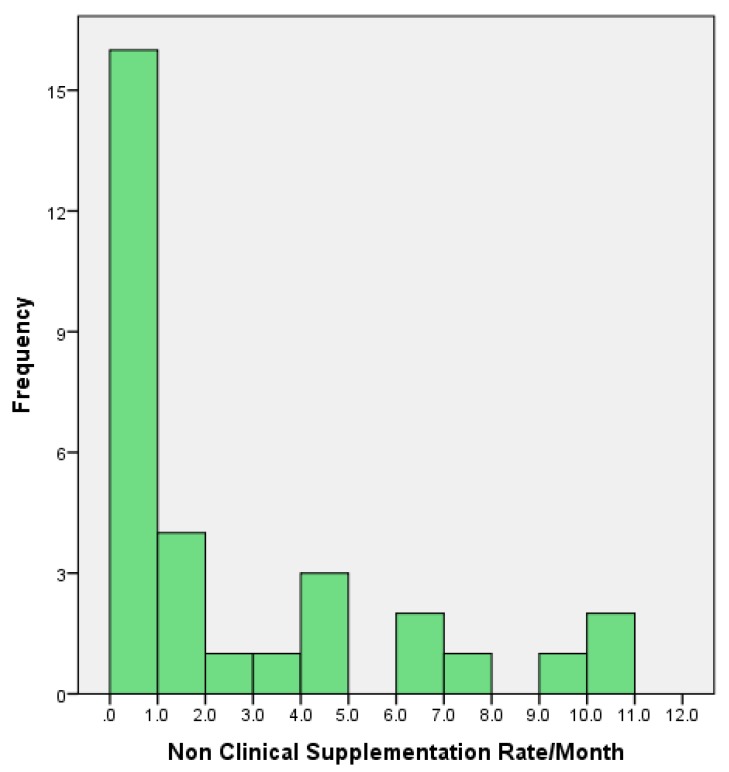
Midwives’ estimated monthly rates of neonatal infant-formula supplementation in the absence of medical indication (NIFS-AMI) as defined by WHO (*n* = 31). Monthly rates were extrapolated from hourly rates. Midwives worked for a median of 212.5 (161.3, 248) hours during the seven-week study period (9 March 2016–26 April 2016).

Figure 2 illustrates the frequency of supplementation behaviour of midwives on the postnatal unit at QCCH. The vast majority of supplements administered during the study period were for non-medically indicated reasons (90%), which was consistent with current literature [48]. Fifty-nine percent of NIFS-AMI instances occurred during between the hours of 22.00–06.00. Medical reasons to supplement included infant hypoglycaemia and very low birth weight (<1500 g). The most commonly recorded reason for NIFS-AMI was maternal request.

### 3.6. What Influences NIFS-AMI?

Table 4 illustrates the univariate analysis of midwife variables in relation to rates of NIFS-AMI. Statistical differences in hourly rates were observed according to ward types (*p* < 0.01), time satisfaction with breastfeeding (*p* = 0.01), and finger feeding support (*p* = 0.02), alongside finger feeding confidence (*p* = 0.03). Borderline significant values were found regarding level of education, perceived impact of BMS supplementation (*p* = 0.07), and the ability to correctly describe three appropriate scenarios (*p* = 0.07).

Univariate analysis according to supplementation categorisation is displayed in Table S2. As a recurring theme, time satisfaction and ward type were significantly associated.

To assess the association between the midwives’ rate of NIFS-AMI and most important exposures, a bootstrapping regression model was used in the multivariate analysis (Table 5). Ward type (*p* = 0.01) and midwife education (*p* = 0.01) were statistically significant.

### 3.7. Perceived Barriers to Supporting Breastfeeding

When asked about barriers encountered on the wards, 78% of the 31 staff members reported time constraints as a key factor affecting breastfeeding support (Figure S2). According to self-reported time allocation, midwives allocated a median of only 12% of their shift to supporting infant feeding, with the largest proportion of their time allocated to computer and paperwork (50%). This was consistent across wards and NIFS-AMI categories.

As a key theme, many midwives expressed emotive responses relating to their perceived lack of time:

“*Sometimes I get depressed, it is a terrible thing not being able to help*”.

Midwife 4

“*The training courses are pointless if you don’t have the time to use it*”.

Midwife 12

“*It’s never them, the mothers want the support; it’s the time*”.

Midwife 30

“*It has repercussions on our own health, not being able to help, as well as of course the baby and the mothers. The mothers get so depressed*”.

Midwife 23

There were also several members of staff expressing the mothers’ poor antenatal preparation as an interfering factor (27.5%). This was expressed in relation to a lack of knowledge and exposure to breastfeeding videos:

“*They [the mothers] genuinely don’t know how good it is! They underestimate the difference between breast and formula, it is such a shame*”.

Midwife 24

“*The videos they show in antenatal classes give women unrealistic expectations of breastfeeding, it is harder than they make it look*”.

Midwife 7

Additionally, workload and staff shortage related issues were common complaints, as reported by 55% of staff. Alongside this, the requirement for more specific staff with dedicated roles to infant feeding and one-to-one opportunities was expressed by 38.5%:

“*You cave in sometimes due to the workload, giving the bottle is so easy and that is why it is so sad*”.

Midwife 23

“*I tell the women breastfeeding is hard and needs perseverance. One-to-one support is so important and 30 min isn’t enough*”.

Midwife 31

“*It is exhausting helping women one-to-one to breastfeed all the time, especially when the ward is busy, it takes a lot of effort*”.

Midwife 18

Although finger feeding was included in the compulsory training day, 38.7% of midwives regarded themselves to have not received training, which was relatively constant across the groups. Time satisfaction and confidence for this technique varied significantly according to ward type. In addition, six staff members (15%) expressed negative attitudes towards finger feeding, describing the practice as “*unhygienic*”, “*dangerous*”, and “*a waste of time*”.

## 4. Discussion

With significant evidence that NIFS-AMI has detrimental consequences on breastfeeding, the factors influencing early BMS use by staff in the post-natal environment is highly relevant. In this study, maternal and midwife factors were sought to determine possible impacts upon NIFS-AMI activity on the postnatal ward.

### 4.1. The Use of BMS Supplementation on the Postnatal Ward

Our data demonstrates that a significant proportion of BMS supplementation occurs for no defined medical reason. In nine out of 10 cases of BMS supplementation, breastmilk substitute was provided by the staff without appropriate medical indication. Local statistics from 2015 Good Food for London Report show a good level of community breastfeeding support, achieving full BFHI accreditation, which further highlights the need to change the current infant feeding practice in the postnatal unit [49]. This recommendation was supported further in the report from the National Maternity and Perinatal Audit 2017, which advised that “Commissioners, together with clinicians, services, and policymakers should strongly prioritise the provision of resources to support breastfeeding, both in maternity units and in the community, to reduce the variation in the proportion of babies receiving breastmilk at their first feed and at discharge from the maternity unit” [50].

### 4.2. Maternal Factors

Maternal characteristics protective against NIFS-AMI were associated with higher educational status, one hour of uninterrupted skin-to-skin after delivery, and breastfeeding discussions during pregnancy with a healthcare professional. These findings are reflective of previous work [51]. The evidence for skin-to-skin as an intervention to improve breastfeeding outcomes is particularly well studied. A 2016 Cochrane review concluded with good evidence that skin-to-skin was beneficial to healthy infants [52]. Our results are supportive of the UNICEF recommendations, which is in agreement with the BFHI 10 steps to successful breastfeeding.

### 4.3. Midwife Factors

Our findings suggest that the staff at QCCH experience many barriers to supporting breastfeeding. One key theme arising from this exploration regards the lack of suitable antenatal preparation. Previous research shows that under-preparation and the availability of BMS are key reasons why mothers requested artificial supplements [53]. In addition, prior expectations, formed from healthcare staff and the media, influence maternal breastfeeding confidence to a major extent [54].

As described by the midwives at QCCH, antenatal classes have been named as partly responsible for these unrealistic expectations [55,56]. This is consistent with the available evidence worldwide, demonstrating antenatal education to have negative or inconclusive impact on breastfeeding rates [57,58,59]. Consequently, a lack of concordance between a mother’s expectations and the reality of breastfeeding can lead to feelings of guilt, failure, and a lack of confidence in breastfeeding and motherhood [60,61].

There have been several studies investigating the factors likely to affect midwives’ behaviour in relation to infant feeding. Lack of time, poor staffing levels, and a resistance to change were all identified as contributing factors to non-compliance of a breastfeeding support intervention on UK postnatal wards [40]. Our data support previous findings in the UK, as the barriers most commonly reported by QCCH maternity staff were a lack of time alongside workload issues and the need for more specific feeding support with one-to-one care. The number of hospitals applying the BFHI policy is on the rise, but the real-life effectiveness of this strategy is somehow limited due to the increasing issue of staff shortages [62,63].

The views of inadequate availability of staff support appear to be consistent across cultural and ethnic backgrounds, with evidence that one-to-one feeding support is effective at significantly reducing supplementation rates [64,65,66]. The perception of active support from midwives postnatally in England was predictive of lower odds of breastfeeding cessation as assessed by maternal questionnaires at day 10 [67]. In this study, many midwives reported computer work, particularly discharges, to take up a considerable percentage of their day. Bowers et al. revealed that very little staff time is spent educating and supporting mothers with infant feeding; this was concordant with the midwives’ time allocation at QCCH. Recent literature has demonstrated a reduction in the duration of postnatal admissions with proportionate reductions in staffing, resulting in an increased workload which negatively impacted quality of care and staff morale [68].

In our study, the most-cited reason for NIFS-AMI was maternal request for BMS. In the literature, reasons for parents requesting BMS supplementation included a lack of preparation for breastfeeding and viewing infant-formula as the answer to breastfeeding problems [24,53].

The UNICEF Maternity Standards document states that clear documentation of the mother’s reason, alternative options, and information provided by midwife must be recorded when BMS supplementation is initiated for maternal requests. The recommended care includes hand expression education, support with positioning, and plans made for future feeding to maximise breastfeeding (or the use of breastmilk) [69]. In reality, this was rarely the case at QCCH; documented notes provided very little details around the initiation of NIFS-AMI. Of the 102 mothers surveyed, only 38% could recall a discussion on hand expressing. The reported midwife dissatisfaction in the workplace appears to be a significant driver in the promotion of breastfeeding and time allocation for appropriate counselling.

As time and workload appear to have significant impacts on patients’ postnatal support, the different working environments may explain the differences reported across ward types [70]. With a lower staff/patient ratio on the NHS postnatal ward, the perceived time satisfaction unsurprisingly differs to those working on private wards or at birth centres. Our study reveals that finger feeding confidence and time satisfaction were negatively associated with higher supplementation rates, which staff attributed to their lack of time. Although time availability appears to play a role in the interference of infant feeding support, a multivariate analysis identified staff perceptions of BMS to be the most influential factor upon rates of NIFS-AMI. This is supported by the inconsistencies identified across shift type and BMS use. For example, many staff reported having more time to support mothers during the night shift; however, a higher frequency of NIFS-AMI instances were reported, suggesting the current practice to be a complex, multifactorial issue.

The next section of the survey was concerned with midwives’ perceptions in relation to supplementation behaviour. Existing evidence suggests that midwives’ behaviour is influential upon a mother’s feeding decisions, with regard to their perceived attitudes and the support offered [71,72,73]. However, the current literature into midwives’ supplementation perceptions and behaviour has not been thoroughly explored. An association between support offered by staff and their attitudes has been shown, with evidence of midwives with poor breastfeeding attitudes failing to support mothers [74]. Mothers usually perceiving maternity staff to have ‘no preference’ with regard to breastfeeding were less likely to breastfeed beyond six weeks [75]. Midwife characteristics associated with greater breastfeeding knowledge in other studies included midwives over 30 years old, with higher qualifications, as well as more clinical and personal experience of breastfeeding for over three months [76,77]. The training a midwife receives has also been suggested to be clinically relevant to breastfeeding practices in the hospital, with those demonstrating higher breastfeeding knowledge reporting best clinical practice [41].

Despite the fact that all maternity staff at QCCH had attended the Trust’s breastfeeding training, only a third of them were able to successfully describe three valid scenarios in which they had supplemented a breastfed infant. This data is in agreement with the outcomes of an American study, reporting high rates of non-medically indicated supplementation, with staff often providing inaccurate information and failing to correct mothers who possessed incorrect information [48]. Although this may indicate that staff lack the knowledge to best advise mothers, there is evidence of non-adherence to the BFHI standards with deviant behaviour described such as undercover supplementation and concealing their actions by recording the supplementation as a ‘maternal choice’ rather than a ‘midwife suggestion’ [78]. This would suggest that midwives may be aware that their behaviour is not the best clinical practice.

### 4.4. Application of BFHI Policy into Clinical Practice

In order to explain the gap between evidence-based knowledge and clinical practice, the knowledge translation theory can be applied to the BFHI policy implementation using five stages from acceptance to adherence [79,80]. Our data demonstrate lack of knowledge as a crucial factor in real-life decision-making on whether to supplement an infant with BMS. In addition, emotional responses expressed by several staff members reflecting personal beliefs on breastfeeding support are consistent with previous qualitative studies in the UK and is thought to have a negative impact on clinical reasoning and adherence to BFHI criteria [81]. It has therefore been suggested that for midwives to develop new behaviour, their attitudes and belief systems need to be reframed [82,83]. This can be particularly difficult to achieve for those who have had positive experiences of supplementing, as beliefs associated with personal involvement most strongly predict forthcoming behaviour [84].

It is evident that for hospital practice modification, active participation and planning for change, alongside establishing targets, are necessary [85]. The midwife-identified barriers, such as those relating to the workplace, require consideration from the establishment to address issues surrounding infant feeding. Making BFHI an organisational priority, reducing the accessibility of BMS, and establishing financial support dedicated to the initiative are all suggestions to overcome barriers from an integrative review [86]. Enforcing the requirement for all parents to read and complete paperwork before BMS is permitted may also be successful at reducing both staff suggested and maternally requested supplementation with greater knowledge communication to ensure that parents give informed consent [87]. This also agrees with earlier results, which shows that support and encouragement from other midwives and managerial staff can promote adherence to BFHI practices, and indicates that involving staff in processes such as mentoring schemes can help promote positive attitudes towards the BFHI [79,87]. Conversely, midwives reverting back to outdated protocols have been demonstrated to influence the behaviour of newly qualified midwives and mothers, highlighting the potential for a cascade of incorrect conduct and the vital requirement for a committed workforce [83,88].

### 4.5. Study Limitations

The main limitations of this study surround the cross-sectional design, with data collection only occurring within a defined point in time. It is therefore impossible to infer causality between outcomes and exposures [89]. A prospective study assessing the attitudes of newly qualified midwives before starting work may therefore provide better insight into the temporal relationship. With limited time available for the interview period, it was not possible to survey all postnatal staff at the QCCH, which could therefore increase the risk of a non-representative sample. Additionally, due to the sample size, only a limited number of confounders could be accounted for in the multivariate analysis. However, the statistically significant correlation between midwives’ education and work placement and the use of NIFS-AMI is worth further investigation. As the survey was presented in English and staff in a single hospital were approached, results cannot be generalised to all medical professionals across the globe. However, we believe that our results are closer to a best-case interpretation and therefore are even more concerning.

## 5. Conclusions

Known modifiable maternal factors confirmed in this study included the protective value of skin-to-skin and antenatal discussions against unnecessary BMS supplementation. A striking number of midwives failing to name medically appropriate scenarios for BMS introduction, which highlights failure in the education system, even when provided in accordance with the latest BFHI guidelines. Available evidence on BFHI effectiveness is somehow conflicting and cannot fully support existing training in its present form. The main outcome of BFHI efficiency is normally limited to breastfeeding initiation rates, which has been continuously critisised as an inappropriate measure to measure the success of the BFHI [90,91].

With an association between the lack of knowledge and greater rates of NIFS-AMI, this study confirms the necessary requirement to reduce in-hospital supplementary feeds, through the development of a better model of breastfeeding support, which may include an extensive use of cell phone-based applications, educational programme development, and postnatal staff training improvement. Midwife interviews in this study suggested a need for more one-to-one support of women in the immediate postnatal period. Maternal request for BMS is a major factor influencing non-clinical supplementation and this should be tackled by developing strategies to train staff to respond constructively to such requests as well as by a more effective education of pregnant women, using online resources and distance learning tools. If changes could be successfully implemented, a reduction in non-clinical supplementation, with regard to both staff suggestion and maternal requests, could be achieved with predicted benefits in breastfeeding outcomes.

Key Messages:

- The use of in-hospital breastmilk substitute supplementation for breastfed infants is commonplace in the study hospital, as 95 (28%) of infants received a BMS supplement prior to discharge from maternity unit and 90% of BMS supplementation was given without medical indication.
- Maternal factors protective of neonatal infant-formula supplementation in the absence of medical indication included skin-to-skin and antenatal breastfeeding discussions.
- Although midwives were appropriately trained in accordance with BFHI protocols, they identified a number of workplace barriers in breastfeeding support, with lack of time and heavy workload being the major contributors.
- We found significant associations between the midwives’ decisions on non-medical infant-formula supplementation and educational status, which suggests a need to focus the existing BFHI training programme.
- In order to reduce unnecessary supplementation as well as address attitudes and perceived barriers, additional educational programmes should be developed, with a focus on one-to-one support for establishing lactation in the postnatal environment.

## Figures and Tables

**Table 1:** Clinical and demographic characteristics of mothers and infants.

	Survey	QCCH	SMH	Both Sites
	*n* = 102	*n* = 486	*n* = 329	*n* = 815
Maternal age (years)	32 (28, 36)	32 (29, 36)	33 (28, 36)	33 (29, 36)
Feeding status		*n* = 220 *	*n* = 117 *	*n* = 337 *
Exclusively BF	55 (54)	138 (63)	75 (64)	213 (63)
Supplemented with medical justification	3 (3)	67 (30)	28 (24)	95 (28)
Supplemented without medical justification				
NIFS-AMI	38 (37)			
Exclusive BMS	6 (6)	15 (7)	14 (12)	29 (9)
Baby age (days)	1 (1, 2)			
Length of admission (days)		2 (1, 3)	2 (1, 4)	2 (1, 3)
Age left education (years)	23 (21, 24)			
Highest level of education: University degree	66 (65)			
Ethnicity: White Caucasian	53/88 (60)	149/295 (51)	106/182 (58)	255/477 (53)
Vaginal delivery	64 (63)	357 (73)	201 (61)	558 (68)
Laboured <2 h	82 (80)	410 (84)	280 (85)	690 (85)
Primiparous	59 (58)	83 (17)	42 (13)	125 (15)
Weight of infant	3.2 (2.9, 3.6)	3.2 (2.9, 3.5)	3.3 (3.0, 3.6)	3.3 (2.9, 3.6)
*Low (<2.5 kg)*	8 (8)	41 (8)	20 (7)	61 (8)
Gestational age (weeks)	39 (38, 40)	39 (38, 40)	39 (39, 40)	39 (38, 40)
If multiparous, EBF previous infants	26/43 (61)			
Ward type				
*Postnatal*	82 (80)	406 (84)	216 (66)	622 (76)
*Private Ward*	9 (9)	0 (0)	70 (21)	70 (9)
*Birth Centre*	11 (11)	80 (16)	43 (13)	123 (15)
Private room	25 (25)			
Infant remained with mother in hospital	92 (90)			
Partner present at night	42 (41)			
Any skin-to-skin after birth	84 (82)			
Skin-to-skin for 1 h after birth	50 (49)			
Supported to BF after birth	68 (67)			
Assisted with positioning and attachment	82 (80)			
Reports having received education about signs that infant is receiving enough milk	50 (49)			
Reports having received education about hand expression	39 (38)			
Reports having antenatal BF discussion with HCP	39 (38)			

All data are displayed as number (% or IQR) as shown. Single numerical values (*n*) in brackets indicate %, double numerical values (*n*, *n*) indicate IQR. Ethnic groups represented in the non-Caucasian sample were categorised according to National Statistics recommended criteria and were represented as follows; surveyed mothers: Black 13 (15), Asian 22 (25), Mixed 0 (0); QCCH: Black 50 (17), Asian 80 (27), Mixed 16 (5); SMH: Black 25 (14), Asian 37 (20), Mixed 14 (8). BF—breastfeeding; BMS—breastmilk substitute; EBF—exclusive breastfeeding; HCP—healthcare professional; NIFS-AMI—neonatal infant-formula supplementation in the absence of medical indication; QCCH—Queen Charlotte’s and Chelsea Hospital; SMH—Saint Mary’s Hospital. * percentages in the corresponding columns were calculated on the available data for statistical analysis.

**Table 2:** Association between maternal characteristics and NIFS-AMI: univariate analysis (*n* = 96).

	Number of Infants Receiving NIFS-AMI	Number of Infants EBF + Medically Justified Supplementation	*p* Value
	*n* = 38	*n* = 55 + 3	
Maternal age (years)	29 (25, 37)	34 (29, 36)	0.13
Baby age (days)	2 (1, 3)	1 (1, 1)	**0.01**
Age left education (years)	22 (19, 24)	23 (21, 24)	0.19
Highest level of education: University degree	18 (47)	44 (76)	**<0.01**
Employed prior to pregnancy	21 (55)	43 (74)	0.08
Ethnicity: White Caucasian	23 (61)	33 (57)	0.29
Vaginal delivery	23 (61)	38 (66)	0.62
Laboured >2 h	31 (82)	45 (78)	0.64
Primiparous	24 (63)	32 (55)	0.44
Weight of infant	3.3 (2.8, 3.6)	3.3 (3.0, 3.5)	
Gestational age (weeks)	39 (38, 40)	40 (39, 41)	0.07
If multiparous, EBF previous infants	6/14 (43)	19/26 (73)	0.06
BF duration with previous infants	12 (5, 24)	7 (6, 9)	0.39
Ward type			**0.04**
*Postnatal*	34 (45)	42 (55)	
*Birth Centre/Private Ward*	4 (25)	16 (75)	
Private room	7 (18)	18 (31)	0.17
Infant remained with mother in hospital	36 (95)	52 (90)	0.47
Partner present at night	18 (47)	20 (35)	0.13
Any skin-to-skin after birth	26 (68)	54 (93)	**<0.01**
Skin-to-skin for 1 h after birth	13 (34)	36 (62)	**0.01**
Supported to BF after birth	27 (71)	41 (71)	0.97
Assisted with positioning and attachment	33 (87)	47 (81)	0.46
Mother reported antenatal education about signs that infant is receiving enough milk	22 (58)	25 (43)	0.16
Mother reported antenatal education on responsive feeding	10 (26)	33 (57)	0.10
Mother reported antenatal education about hand expression	23 (61)	15 (26)	**<0.01**
Mother reported antenatal education on benefits of developing relationship with infant	14 (37)	32 (55)	**0.01**
Informed of support available within the community	17 (45)	24 (41)	0.75
Attended BF antenatal class at QCCH	3 (8)	7 (12)	0.74
Attended BF antenatal class at another site	7 (18)	16 (28)	0.30
Antenatal BF discussion	9 (24)	29 (50)	**0.01**
Mother reports having been EBF as infant	16 (43)	23 (40)	0.99
Duration EBF as infant	12 (12, 24)	7 (6, 12)	**0.05**
BF confidence on ward (1–10)	7 (6, 8)	7 (6, 9)	0.28
BF confidence before birth (1–10)	8 (6, 10)	6 (5, 9)	0.09
BMS first introduced (hours)	6 (4, 24)	5 (3, 39)	0.94
Positive attitude to BF in social/cultural background	29 (76)	46 (79)	0.73

All data are displayed as number (% or IQR) as shown. Single numerical values (*n*) in brackets indicate %, double numerical values (*n*, *n*) indicate IQR. Mothers exclusively feeding their infants with BMS have been excluded from the multivariate analysis (*n* = 6). Significant results are indicated by the bold text. BF—breastfeeding; BMS—breastmilk substitute; EBF—exclusively breastfed; NIFS-AMI—neonatal infant-formula supplementation in the absence of medical indication; QCCH—Queen Charlotte’s and Chelsea Hospital.

**Table 3:** Midwife attitude and knowledge of breastfeeding.

Midwife Attitude and Knowledge of Breastfeeding	*n* (IQR)
Perceived importance of role in BF (1–10) *	10 (10, 10)
Perceived impact of BMS supplements (1–10) *	8 (6, 10)
Perceived time satisfaction (1–10) *	5 (1, 8)
Confidence in FF (1–10) *	8 (4, 10)
	***n* (%)**
Positive attitudes to BF in social/cultural background	24 (77)
Staff satisfied with time for FF	10 (32)
Received training on FF	19 (61)
Supplementation practice violates WHO criteria	20 (65)
One of three examples violated criteria	6 (19)
Two of three examples violated criteria	10 (32)
All examples described violated criteria	4 (13)
Percentage of time allocation to feeding support versus other tasks during a typical day	**% (IQR)**
Specific feeding support	12 (10, 20)
General patient care	25 (20, 40)
Computer/paperwork	50 (30, 60)
Taking bedside observations	10 (10, 15)

BF—breastfeeding; BMS—breastmilk substitute; FF—finger feeding; WHO—World Health Organisation. *—Likert scale was used to assess these items.

**Table 4:** Hourly rates of NIFS-AMI (%) according to midwife variables (*n* = 31).

Descriptive Characteristics	Median/IQR	r	*p* Value
Staff age		0.2	0.28
Years’ experience as a midwife		0.03	0.89
**Ward Type**			
*Postnatal*	1.9 (0.7, 4.2)		**<0.01**
*Birth Centre/Private Ward*	0.0 (0.0, 0.4)		
Ethnic Background			
*White Caucasian*	0.4 (0.0, 0.7)		0.26
*Non-Caucasian*	1.9 (0.6, 3.0)		
Highest level of education			
*Degree*	0.4 (0.0, 1.9)		0.07
*Non-degree*	3.4 (2.2, 4.7)		
**Personal experiences**			
Attitudes to BF in social/cultural background			
*Positive*	0.7 (0.0, 2.7)		0.84
*Negative or mixed*	0.4 (0.0, 2.7)		
Parity			
*Primiparous/multiparous*	0.7 (0.4, 4.2)		0.22
*Nulliparous*	0.4 (0.0, 1.8)		
Infant feeding method			
*EBF*	0.6 (0.0, 1.9)		0.57
*Non-EBF*	2.5 (0.5, 4.2)		
Longest BF duration (months)		−0.29	0.32
Earliest age child received non-breastmilk (months)		−0.79	0.58
Midwife’s reported feeding method as infant			0.62
*EBF*	0.6 (0.0, 1.8)		
*Mon-EBF*	0.8 (0.0, 2.7)		
Duration of BF as infant (months)		0.43	0.11
**Staff perceptions and professional experiences**			
Perceived impact of BMS supplementation (1–10)		−0.33	0.07
Happiness with time for infant feeding support (1–10)		−0.47	**0.01**
Attended training sponsored by a formula company			
*Yes*	0.8 (0.3, 2.7)		0.62
*No*	0.5 (0.0, 2.6)		
Received training on finger feeding			
*Yes*	0.4 (0.0, 3.0)		0.86
*No*	0.9 (0.0, 2.7)		
Satisfied with time available for finger feeding			
*Yes*	0.0 (0.0, 0.6)	0.43	**0.02**
*No*	1.2 (0.3, 4.2)		
Confidence in finger feeding (1–10)		−0.40	**0.03**
Correctly identify three correct reasons for supplementing			
*Yes*	0.6 (0.0, 4.6)		0.07
*No*	0.6 (0.0, 2.1)		
Percentage of time allocation to feeding support versus other tasks during a typical day (%)			
*Infant feeding support*		0.56	0.77
*General patient care*		−0.01	0.96
*Computer/paperwork*		−0.01	0.95
*Taking bedside observations*		0.14	0.47

r—Spearman Rho or Pearson’s correlation coefficient. Mann-Whitney U, Spearman Rho, and Pearson’s coefficient were used for categorical, non-parametric, and parametric correlations, respectively. Significant results are indicated by the bold text. BF—breastfeeding; BMS—breastmilk substitute; EBF—exclusively breastfed; NIFS-AMI—neonatal infant-formula supplementation in the absence of medical indication.

**Table 5:** Multivariate analysis of factors influencing midwives’ decision for NIFS-AMI.

Parameter	B	*p* Value	95% Confidence Interval	
			Lower Bound	Upper Bound
Education	2.53	**0.01**	0.95	4.12
Breastmilk substitute impact (1–10)	−0.19	0.22	−0.48	0.07
Ability to provide three correct scenarios (100%)	1.01	0.18	−2.47	0.23
Ward	−1.63	**0.01**	−2.50	−0.66

Dependent variable; rate of NIFS-AMI (%), a; this parameter is set to zero because it is redundant. Significant results are indicated by the bold text.

## Supplementary Materials

The following are available online at http://www.mdpi.com/2072-6643/10/5/608/s1, Figure S1: The monthly rates of exclusive breastfeeding at discharge for 2016, Figure S2: Staff’s perceived barriers to supporting breastfeeding on the wards, Table S1: Midwife characteristics, Table S2: Univariate analysis according to supplementation categorisation.
