# Supplementary material for: Formula Milk Supplementation on the Postnatal Ward: A Cross-Sectional Analytical Study
Source: Nutrients. 2018 May 14;10(5):608. doi: 10.3390/nu10050608 (PMC5986488; doi:10.3390/nu10050608)
Supplement: Supplementary file 1 [file nutrients-10-00608-s001.pdf]

Supplementary Materials

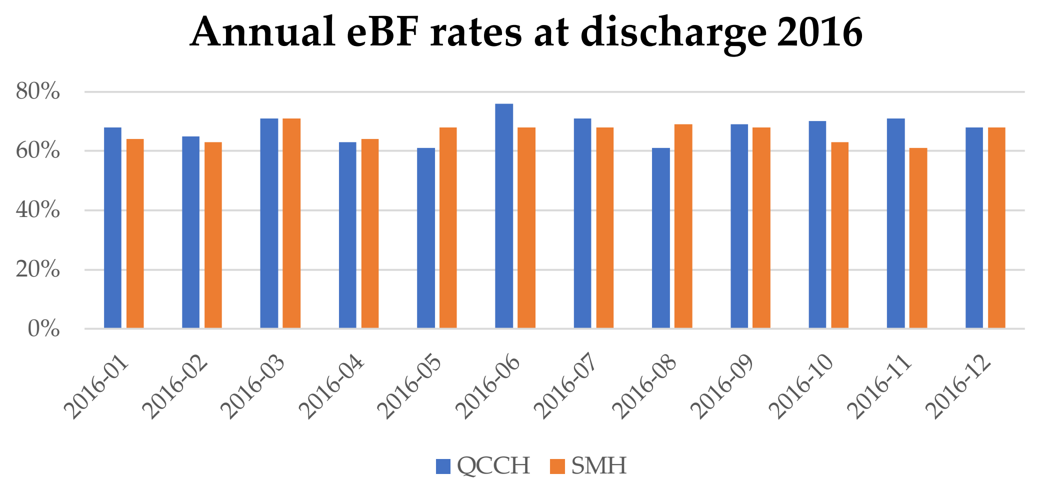

Figure S1: Annual eBF rates at discharge on the postnatal wards at QCCH and SMH in 2016. Percentages (%) are based on the feeding methods recorded at discharge of 4388 infants. Monthly eBF rates for QCCH and SMH are shown. The overall annual eBF rate was 67%. QCCH; Queen Charlotte's and Chelsea Hospital, SMH; Saint Mary's Hospital: eBF; exclusively breastfed. Clinical supplementation: 78 (58.7%) of non-clinical supplementation occurred between the hours of 22.00-06.00. Clinical reasons to supplement included infant hypoglycaemia and very low birth weight (<1500g). Most commonly recorded supplementation reason was maternal request.

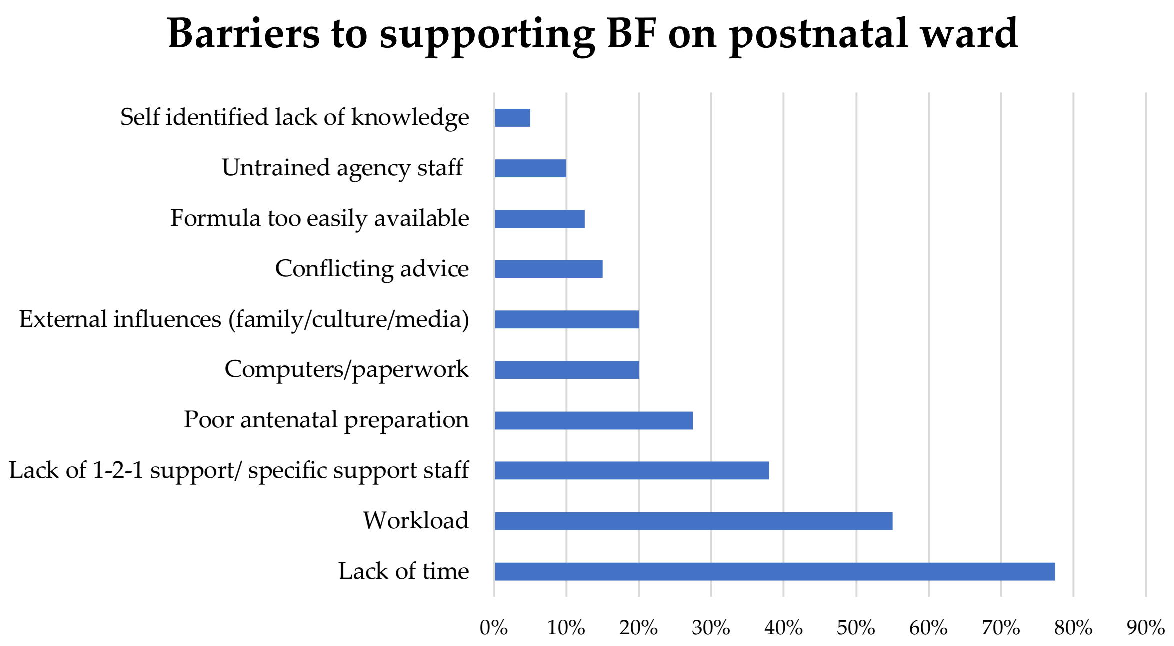

Figure S2: Staff's perceived barriers to supporting breastfeeding on the wards. Percentage (%) of total staff (n=31) expressing each theme is graphically represented.

Table S1: Midwife characteristics

**Midwife characteristics**	
Staff age	38 (28, 50)
Years' experience as midwife	7 (4, 18)
Ward Type	
Postnatal	18 (58)
Birth Centre/Private ward	13 (42)
Ethnicity: White Caucasian	22 (71)
Educated to degree level	27 (87)
NIFS-AMI	20 (65)
Non-clinical supplementation rate (%)	0.6 (0.0, 2.8)
Attended training sponsored by a formula company	9 (29)
Primiparous/multiparous	14 (45)
eBF own infants	8 (57)
Longest BF duration (months)	14 (5, 23)
Earliest age child received non-breast milk (months)	3 (1,6)
eBF as infant	17 (57)
Duration of BF as infant (months)	6 (4, 12)

All data are displayed; number (% or IQR) as shown. Ethnic groups represented in the non- Caucasian midwives were Black African 8 (25.8) and Asian 1 (3.2). 100% staff attended BF training course within the Imperial College London NHS Trust. BF-breastfeeding; eBF –exclusively breastfeeding: NIFS-AMI-neonatal infant-formula supplementation in the absence of medical indication.

Table S2: Midwife characteristics according to their supplementation categorisation (*n* = 31).

	**Supplementing midwives *n* = 20**	**Non-supplementing midwives *n* = 11**	***p* =**
**Descriptive characteristics**			
**Staff age**	41 (28, 56)	30 (28, 39)	**0.044**
Years' experience as midwife	8 (4, 23)	5 (4, 9)	0.212
**Ward Type**			
Postnatal	15 (75)	3 (27)	**0.02**
Birth Centre/ Private Ward	5 (25)	8 (73)	
Ethnicity: White Caucasian	12 (60)	10 (91)	0.11
Educated to degree level	16 (80)	11 (100)	0.269
**Personal experiences**			
Positive attitudes to BF in social/cultural background	16 (80)	8 (73)	0.676
Primiparous/multiparous	11 (55)	3 (27)	0.26
eBF own infants	5 (46)	3 (100)	0.209
Longest BF duration (months)	12 (4, 16)	24 (16, 27)	0.29
Earliest age child received non-breast milk (months)	2 (1, 5)	4 (3, 5)	0.456
eBF as infant	10 (50)	7 (64)	0.708
Duration of BF as infant (months)	11 (5, 12)	6 (3, 6)	0.17
**Staff perceptions and professional experiences**			
Perceived impact of BMS (1-10)	7 (5, 9)	8 (7, 10)	0.183
Time satisfaction for infant feeding support (1-10)	3 (1, 6)	6, 3, 9)	0.032
Attended training sponsored by a formula company	6 (30)	2 (18)	0.429
Received training on finger-feeding	12 (60)	7 (64)	1

Satisfied with time available for finger-feeding	4 (20)	6 (55)	0.106
Confidence in finger-feeding (1-10)	7 (2, 10)	10 (8 10)	0.07
Correctly identify three correct reasons for supplementing	7 (35)	4 (36)	1
Time allocation by percentage			
Computer/paperwork	45 (24, 60)	50 (30, 60)	0.594
General patient care	25 (20, 33)	30 (20, 40)	0.729
Infant feeding support	12 (10, 20)	10 (10, 20)	0.945
Observations and medication	11 (10, 20)	10 (5, 10)	0.077

Data is shown as median (percentage or interquartile range). Mann-Whitney U Test and Fisher's Exact Test (2-tailed) were used. BMS-breast milk supplements; BF-breastfeeding; eBF –exclusively breastfeeding: NIFS-AMI- neonatal infant-formula supplementation in the absence of medical indication.
